# Association of dietary magnesium intake and glycohemoglobin with mortality risk in diabetic patients

**DOI:** 10.1371/journal.pone.0277180

**Published:** 2022-12-28

**Authors:** Hung-Wei Wang, Yun-Ting Huang, Ming-Yan Jiang

**Affiliations:** 1 Renal Division, Department of Internal Medicine, Chi Mei Hospital Chiali, Tainan, Taiwan; 2 Renal Division, Department of Internal Medicine, Chi Mei Medical Center, Tainan, Taiwan; 3 Department of Pharmacy, Chia Nan University of Pharmacy & Science, Tainan, Taiwan; SUNY Oneonta, UNITED STATES

## Abstract

**Background:**

Dietary magnesium intake inversely correlated to risk of death in general population. However, it is relatively unknown whether the beneficial effect remains significant in individuals with diabetes. Our study purpose is to evaluate the association of dietary magnesium intake with mortality risk in diabetic population.

**Methods:**

The study population is recruited from 2003–2014 National Health and Nutrition Examination Survey, totaling 2,045 adults with diabetes being included. Participants were divided based on glycohemoglobin (HbA1c < 7% and ≥ 7%) and daily dietary magnesium intake (≤ and > 250mg/day) ascertained by 24-hour dietary recall interviews.

**Results:**

The average age of the study population was 52.9±10.1 years, with 49.1% being male. During a median follow-up of 77.0 months (interquartile range: 45.0–107.0 months), a total of 223 participants died (1.5 per 1000 person-months). Our results showed that individuals with lower dietary magnesium intake (≤250mg/day) had higher risk of all-cause (HR: 1.56, 95% CI: 1.13–2.16) and other-cause (non-cardiovascular and non-cancer) mortality (HR: 1.68, 95% CI: 1.09–2.60), while cardiovascular and cancer-related mortality were similar compared with individuals with magnesium intake > 250mg/day. We also showed that the risk of all-cause (HR: 1.86, 95% CI: 1.33–2.60) and other-cause mortality (HR: 2.03, 95% CI: 1.29–3.19) were higher in individuals with poorly controlled diabetes (HbA1c ≥7.0%) compared with HbA1c <7.0%; however, the association attenuated in the subgroup of higher magnesium intake (>250mg/day). When combining HbA1c and dietary magnesium intake, we showed that individuals with HbA1c ≥ 7% and dietary magnesium intake ≤ 250 mg/day had higher all-cause and other-cause (non-cardiovascular and non-cancer) mortality risk compared with those with HbA1c < 7% and/or dietary magnesium intake > 250 mg/day.

**Conclusion:**

Higher magnesium intake may help reduce mortality risk in individuals with diabetes and attenuate mortality risk of poor diabetic control.

## Introduction

Diabetes mellitus imposes a heavy burden on public health and is one of the leading causes of death globally [1], accounting for 4.2 million deaths in adults aged 20–79 years in 2019 [2]. Studies have shown that poor control of diabetes correlated to several adverse health outcomes including cardiovascular disease (CVD) and all-cause mortality [3–5]. When compared to those with glycohemoglobin (HbA1c) < 6.5%, diabetic patients with HbA1c > 7.4% had higher all-cause mortality risk [5, 6].

Magnesium deficiency is associated with insulin resistance and impairment of cellular glucose uptake [7], while a diet with higher magnesium correlates to reduced risk of incident type 2 diabetes and metabolic syndrome [8, 9]. Among general population, several studies have demonstrated the beneficial effect of higher magnesium intake in reducing CVD and all-cause mortality risk [9, 10], while lower dietary magnesium correlated to increased risk of cardiovascular, cancer, and all-cause mortality [11]. Additionally, among a population with high cardiovascular risk with nearly 50% having diabetes, a previous study showed that higher dietary magnesium was associated with lower all-cause mortality risk [12]. In individuals with type 2 diabetes, a meta-analysis suggested that oral magnesium supplementation may reduce fasting plasma glucose and raise high-density lipoprotein cholesterol levels, although long-term beneficial effect remained to be determined [13].

Despite the vigorous evidence of the beneficial effect of magnesium intake on mortality risk in general population, evidence in individuals with diabetes is relatively lacking. Accordingly, our study purpose is to evaluate the association of dietary magnesium intake on mortality risk among diabetic population. We also investigate whether higher dietary magnesium intake correlates to reduced long-term mortality among poorly controlled diabetes.

## Methods

### Data source

Our data is obtained from National Health and Nutrition Examination Survey (NHANES) of the United States (U.S.), which is a series of health-related programs conducted by the National Center for Health Statistics [14]. NHANES constitutes a series of cross-sectional, multistage probability sampling for civilian noninstitutionalized population across the U.S. (https://wwwn.cdc.gov/nchs/nhanes/tutorials/module2.aspx). NHANES data are collected from survey participants using questionnaires on health-related topics in participants’ homes and a physical examination and laboratory tests in a mobile examination center, with data release in 2-year cycles. The data were available for public use on the website of National Center for Health Statistics. (Avail-able from: https://www.cdc.gov/nchs/nhanes/index.htm.). All NHANES protocols were approved by the research ethics review board of the National Center for Health Statistics, and all the participants provided written informed consent.

### Study population

**Fig 1:**
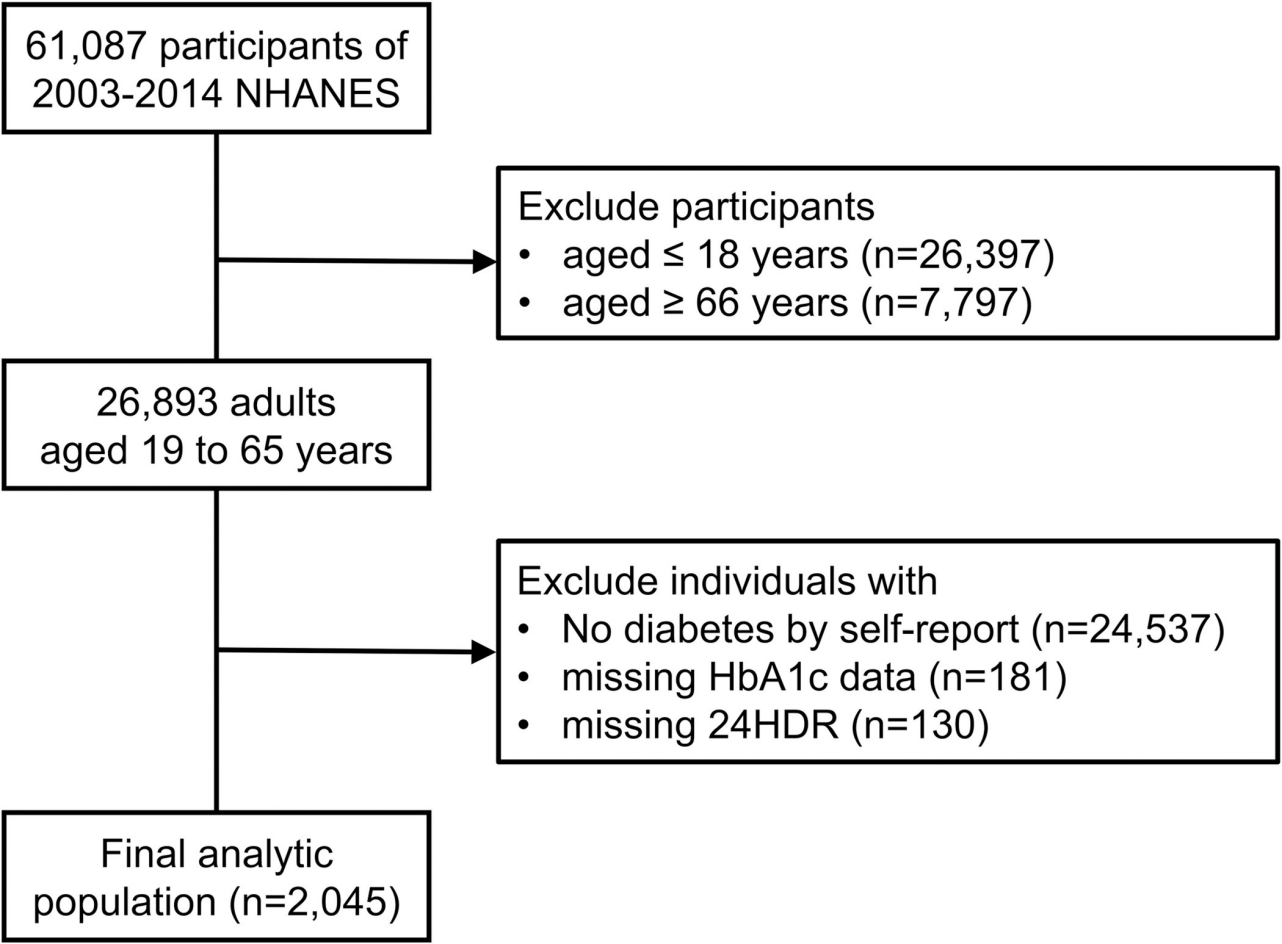
Flow diagram of the study population selection. NHANES: National Health and Nutrition Examination Survey; HbA1c: glycohemoglobin; 24HDR: 24-hour dietary recall.

In this retrospective cohort study, we merged the data from 6 discrete 2-year cycles (2003–2004 through 2013–2014) of the continuous NHANES (N = 61,087). We restricted our study population to 2003 through 2014 NHANES cycles to avoid prolonged time frame of enrollment to reduce the probability of repeated measurements of participants and period effect due to medical advance. We included individuals aged 19 to 65 years at the time of examination with diabetes mellitus (n = 2,356), which was defined by self-reported being diagnosed with the disease or taking medications, either diabetic pills or insulin. We precluded individuals without HbA1c data (n = 181) or 24-hour dietary recall interviews (n = 130). The final analytic population consisted of 2,045 participants (Fig 1).

### Exposure

Dietary intake data were collected through 24-hour dietary recall interviews, which were conducted in-person in the Mobile Examination Center. The dietary intake data are used to estimate the types and amounts of foods and beverages consumed during the 24-hour period prior to the interview (midnight to midnight), and to estimate intakes of energy, nutrients, and other food components from those foods and beverages. Dietary magnesium intake was ascertained from the Total Nutrient Intakes files, which provide a summary record of total nutrient intakes. A validation study had revealed that using 24-hour dietary recall underestimated minerals intake by an average of 10% among adult participants of NHANES [15]. In this study, we dichotomized the participants by dietary magnesium intake into groups of ≤ 250 mg/day (n = 1,062) and > 250 mg/day (n = 983), a level accounting for the underestimation of 24-hour dietary recall and to provide balanced numbers of participants in each group. We also divided the participants by HbA1c < 7.0% (n = 965) and ≥ 7.0% (n = 1,080) to provide balanced numbers of participants in each group.

### Outcome

The NHANES data are linked to death records from the National Death Index (NDI) to ascertain survival status through probabilistic matching and death certificate review. International Classification of Diseases–Ninth Revision were used to define the cause of death prior to 1999 and International Classification of Diseases–Tenth Revision codes for deaths from 1999 to 2015. Deaths due to numerous causes were identified according to the leading causes of death included in the publicly available NHANES linked mortality file. We classified the causes of death as CVD (I00-I09, I11, I13, I20-I51), cancer (C00-C97), and others (non-CVD, non-cancer). The follow-up period for each participant is the time period between the NHANES baseline examination date and the participant’s death date or last date of follow-up (December 31, 2015), whichever came first. At the time of our study, the NDI data linkage was available for 1999–2000 through 2013–2014 NHANES cycles.

### Covariates

Race/ethnicity was categorized as non-Hispanic Whites, non-Hispanic Blacks, Hispanics, and other race including multi-racial by self-report. Ratio of family income to poverty (PIR) was calculated by dividing total family income by the poverty threshold specific to family size and the appropriate year and state. We classified PIR into 3 categories: <1.3 (low income), ≥1.3 to < 3.5 (middle income), and ≥3.5 (high income). Marital status was dichotomized into married or living with partner, and widowed, divorced, separated, or never married. Educational level was dichotomized into ≤ high school graduate and some college or above. Body mass index (BMI) was calculated as body weight in kilograms divided by the square of height in meters. Hypertension was defined by self-reporting diagnosis with the disease or taking medications. Cardiovascular disease (CVD) was defined by self-reported history of congestive heart failure, coronary heart disease, angina, or heart attack; previous stroke was also defined by self-reported his-tory of the diseases. The estimated glomerular filtration rate (eGFR) was calculated by Chronic Kidney Disease Epidemiology Collaboration 2021 (CKD-EPI 2021) equation [16]. Albuminuria was defined as urinary albumin creatinine ratio (ACR) ≥ 30 mg/g.

### Statistical analysis

Continuous variables were presented as mean ± standard deviation and were tested by independent sample t test. Categorical variables were presented as numbers (percent) and were compared by χ^2^ tests. Tests were two-tailed with a significance level of 0.05. We performed Kaplan-Meier method with Log-Rank test to plot the survival curves. Cox regression analysis was performed to explore the association between dietary magnesium intake and mortality risk with adjustment for age, sex, self-identified race/ethnicity, BMI, albuminuria, eGFR, HbA1c, hypertension, CVD, previous stroke, smoking status, marital status, educational level, and ratio of family income to poverty. We also performed Cox regression analysis and stratified by dietary magnesium ≤ 250 mg/day or > 250 mg/day to evaluate the association of dietary magnesium and HbA1c with mortality risk (with adjustment of age, sex, self-identified race/ethnicity, BMI, albuminuria, eGFR, hypertension, CVD, previous stroke, smoking status, marital status, educational level, and ratio of family income to poverty). We further combined HbA1c and dietary magnesium intake and divided the participants into four groups. Group 1 were those with HbA1c ≥ 7.0% with dietary magnesium intake ≤ 250 mg/day (n = 516), group 2 were those with HbA1c ≥ 7.0% with dietary magnesium intake > 250 mg/day (n = 564), group 3 were those with HbA1c < 7.0% with dietary magnesium intake ≤ 250 mg/day (n = 467), and group 4 were those with HbA1c < 7.0% with dietary magnesium intake > 250 mg/day (n = 498). We performed Cox regression analysis with adjustment for age, sex, self-identified race/ethnicity, BMI, albuminuria, eGFR, hypertension, CVD, previous stroke, smoking status, marital status, educational level, and ratio of family income to poverty to examine the mortality risk among the 4 groups. Data were presented as hazard ratio (HR) and 95% confidence interval (CI). The proportional hazards assumption was tested by including time-dependent covariates in the Cox model and showed no violation of the assumption. Because the recommended dietary intake of magnesium was 300~400 mg/day, we also performed the analyses with cutoff levels of 350 mg/day for dietary magnesium intake and 6.5% for HbA1c and the results were provided in the supplementary tables. Statistical computation was performed using SAS 9.4.

## Results

The average age of the study population was 52.9±10.1 years old and 49.1% of them was male (Table 1), with race/ethnicity distribution of 32.0% Whites, 29.5% Blacks, and 31.5% Hispanics. Individuals with higher dietary magnesium intake (>250 mg/day) tended to be male, married or living with partner, higher in educational level, higher in family income, lower in comorbidities including hypertension, CVD, and previous stroke, higher in eGFR, and higher in triglyceride (TG). There was no difference in average age, BMI, prevalence of albuminuria, and HbA1c (Table 1).

**Table 1:** Baseline characteristics of the study population.

	Total (N = 2,045)	dietary Mg ≤ 250 mg/day (N = 983)	dietary Mg > 250 mg/day (N = 1062)	*p* value
Age (years old)	52.9±10.1	53.1±10.2	52.7±10.1	>0.05
Male	1005 (49.1%)	361 (36.7%)	644 (60.6%)	<0.001
Race				<0.001
Whites	655 (32.0%)	279 (28.4%)	376 (35.4%)	
Black	604 (29.5%)	350 (35.6%)	254 (23.9%)	
Hispanics	644 (31.5%)	296 (30.1%)	348 (32.8%)	
Others	142 (6.9%)	58 (5.9%)	84 (7.9%)	
Educational level				<0.001
≤ high school	1141 (55.8%)	609 (62.0%)	532 (50.1%)	
≥ some college	902 (44.2%)	373 (38.0%)	529 (49.9%)	
Marital status				<0.001
Married or living with partner	1244 (61.1%)	558 (56.9%)	686 (65.1%)	
widowed/divorced/separate/never married	791 (38.9%)	423 (43.1%)	368 (34.9%)	
Ratio of family income to poverty				<0.001
<1.3	715 (37.8%)	404 (44.4%)	311 (31.6%)	
1.3 to < 3.5	680 (35.9%)	318 (35.0%)	362 (36.8%)	
≥ 3.5	499 (26.3%)	187 (20.6%)	312 (31.7%)	
BMI (kg/m2)	33.6±8.0	33.8±8.2	33.4±7.9	>0.05
BMI < 25	223 (11.1%)	103 (10.7%)	120 (11.4%)	
BMI 25 to < 30	516 (25.7%)	237 (24.7%)	279 (26.6%)	
BMI ≥ 30	1271 (63.2%)	621 (64.6%)	650 (62.0%)	
SBP	127.8±19.1	127.8±19.8	127.8±18.5	>0.05
DBP	71.9±11.8	71.3±11.8	72.5±11.7	<0.05
Smoking status				<0.001
Never smoker	1018 (50.0%)	497 (50.8%)	521 (49.2%)	
Former smoker	563 (27.6%)	235 (24.0%)	328 (31.0%)	
Current smoker	456 (22.4%)	246 (25.2%)	210 (19.8%)	
Hypertension	1348 (65.9%)	671 (68.3%)	677 (63.7%)	<0.05
CVD	354 (17.4%)	207 (21.1%)	147 (13.9%)	<0.001
Old stroke	138 (6.8%)	87 (8.9%)	51 (4.8%)	<0.001
eGFR	90.2±23.8	87.3±26.2	92.8±21.1	<0.001
Albuminuria	603 (30.1%)	305 (32.0%)	298 (28.3%)	>0.05
TG (mg/dL)	210.9±241.7	197.7±174.0	223.0±290.1	<0.05
Cholesterol (mg/dL)	191.9±50.2	193.9±49.1	190.0±51.2	>0.05
Uric acid	5.59±1.56	5.58±1.57	5.60±1.56	>0.05
Glucose (mg/dL)	161.4±81.3	160.2±83.3	162.4±79.5	>0.05
HbA1c (%)	7.60±1.99	7.63±2.05	7.57±1.94	>0.05
HbA1c ≥ 7.0	1080 (52.8%)	516 (52.5%)	564 (53.1%)	>0.05

Mg: magnesium; BMI: body mass index; SBP: systolic blood pressure; DBP: diastolic blood pressure; CVD: cardiovascular disease; eGFR: estimated glomerular filtration rate (ml/min/1.73 m^2^); TG: triglyceride; HbA1c: glycohemoglobin.

**Fig 2:**
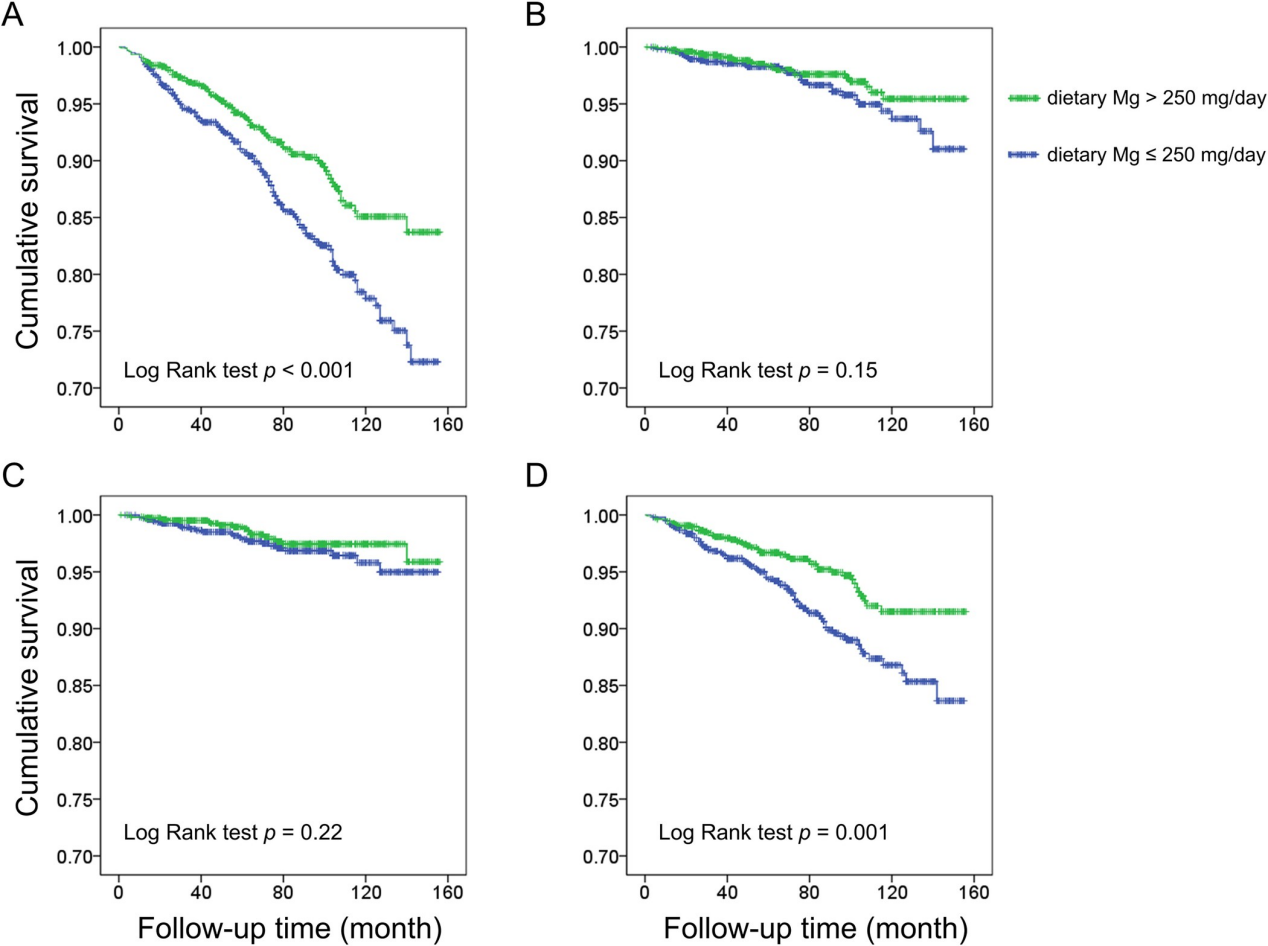
Survival curves for (A) all-cause mortality, (B) cardiovascular disease (CVD) related mortality, (C) cancer related mortality, and (D) other-cause (non-CVD, non-cancer) mortality, by Kaplan-Meier method with Log Rank test. Mg: magnesium.

During a median follow-up of 77.0 months (interquartile range: 45.0–107.0 months), a total of 223 participants died (1.5 per 1000 person-months), of whom 55 died from CVD, 43 died from cancer, and 125 died from other causes. Our results showed that individuals with lower dietary magnesium intake (≤ 250 mg/day) had higher risk of all-cause mortality and other-cause mortality when compared with those with higher dietary magnesium intake (>250 mg/day), while the risks of CVD and cancer-related mortality were not significantly different between the two groups (Fig 2 and Table 2). By Cox regression analysis, we showed that lower dietary magnesium intake (≤ 250 mg/day) is a significant risk factor of all-cause mortality (HR: 1.56, 95% CI: 1.13–2.16, p<0.01) and other-cause mortality (HR: 1.68, 95% CI: 1.09–2.60, p<0.05) after adjusting for age, sex, race, BMI, albuminuria, eGFR, HbA1c, hypertension, cardiovascular disease, previous stroke, smoking status, marital status, educational level, and ratio of family income to poverty (Table 2).

**Table 2:** All-cause and cause-specific mortality risk in individuals with lower dietary magnesium intake (≤ 250 mg/day) compared with higher dietary magnesium intake (> 250 mg/day).

		Crude model				Adjusted model ^#^			
	No. of event	HR	95% CI		*p*	HR	95%CI		*p*
Cause of death			Lower	Upper			Lower	Upper	
All-cause	223	1.66	1.26	2.17	<0.001	1.56	1.13	2.16	<0.01
CVD	55	1.48	0.86	2.52	>0.05	1.45	0.72	2.92	>0.05
Cancer	43	1.46	0.80	2.68	>0.05	1.33	0.66	2.68	>0.05
Other-cause	125	1.82	1.27	2.62	<0.01	1.68	1.09	2.60	<0.05

^#^: adjusted for age, sex, race, body mass index, albuminuria, estimated glomerular filtration rate, HbA1c, hypertension, cardiovascular disease (CVD), previous stroke, smoking status, marital status, educational level, and ratio of family income to poverty.

HR: hazard ratio; CI: confidence interval.

Among total population, we showed that HbA1c ≥ 7.0% is a significant predictor of all-cause mortality (HR: 1.86, 95% CI: 1.33–2.60, p<0.001) and other-cause mortality (HR: 2.03, 95% CI: 1.29–3.19, p<0.01) after adjusting for potential confounders (Table 3).When stratified by dietary magnesium intake, our results showed that HbA1c ≥ 7.0% is still a significant predictor of all-cause mortality (HR: 2.22, 95% CI: 1.41–3.48, p<0.001) and other-cause mortality (HR:2.29, 95% CI: 1.26–4.16, p<0.01) among those with dietary magnesium intake ≤ 250 mg/day. However, the association between higher HbA1c (≥ 7.0%) and risk of all-cause mortality or other-cause mortality attenuated among those with dietary magnesium intake > 250 mg/day (Table 3).

**Table 3:** All-cause and cause-specific mortality risk associated with HbA1c ≥ 7.0% in the total population and in subgroups stratified by daily dietary magnesium (Mg) intake ≤ and > 250 mg/day.

	All-cause mortality HR (95% CI)	CVD mortality HR (95% CI)	Cancer mortality HR (95% CI)	Other-cause mortality HR (95% CI)
Total population				
A1c <7.0%	1	1	1	1
A1c ≥7.0%	1.86 (1.33–2.60) ***	1.49 (0.74–3.00)	1.91 (0.93–3.93)	2.03 (1.29–3.19) **
Dietary Mg ≤250 mg/day				
A1c <7.0%	1	1	1	1
A1c ≥7.0%	2.22 (1.41–3.48) ***	2.56 (0.92–7.10)	1.73 (0.63–4.78)	2.29 (1.26–4.16) **
Dietary Mg >250 mg/day				
A1c <7.0%	1	1	1	1
A1c ≥7.0%	1.50 (0.89–2.51)	0.84 (0.28–2.56)	2.06 (0.68–6.25)	1.71 (0.82–3.54)

The cox regression models were adjusted for age, sex, race, body mass index, albuminuria, estimated glomerular filtration rate, hypertension, cardiovascular disease (CVD), previous stroke, smoking status, marital status, educational level, and ratio of family income to poverty.

*: p<0.05

**: p<0.01

***: p<0.001.

When dividing study participants into 4 groups by HbA1c and dietary magnesium intake, our results showed that group 2 (HR: 0.56, 95% CI 0.38–0.83, p<0.01), group 3 (HR: 0.47, 95% CI 0.30–0.72, p<0.001), and group 4 (HR: 0.38, 95% CI 0.24–0.61, p<0.001) were associated with reduced risk of all-cause mortality compared with group 1 (Table 4). Additionally, group 2 (HR: 0.53, 95% CI 0.32–0.89, p<0.05), group 3 (HR: 0.43, 95% CI 0.24–0.77, p<0.01), and group 4 (HR: 0.34, 95% CI 0.18–0.65, p<0.01) also had lower risk of other-cause mortality compared with group 1. There was no difference in risk of all-cause, CVD, cancer, or other-cause mortality among group 2, group 3, and group 4 (all p values > 0.05). When using cutoff levels of 350 mg/day for magnesium intake and 6.5% for HbA1c, we observed similar results (S1 and S2 Tables).

**Table 4:** All-cause and cause-specific mortality risk among the four groups of study participants by glycohemoglobin (HbA1c) and dietary magnesium intake.

	All-cause mortality HR (95% CI)	CVD mortality HR (95% CI)	Cancer mortality HR (95% CI)	Other-cause mortality HR (95% CI)
Group 1	1	1	1	1
Group 2	0.56 (0.38–0.83) **	0.50 (0.21–1.20)	0.81 (0.34–1.92)	0.53 (0.32–0.89) *
Group 3	0.47 (0.30–0.72) ***	0.50 (0.19–1.33)	0.59 (0.22–1.54)	0.43 (0.24–0.77) **
Group 4	0.38 (0.24–0.61) ***	0.51 (0.20–1.28)	0.37 (0.13–1.09)	0.34 (0.18–0.65) **
*P* for trend	<0.001	>0.05	>0.05	<0.001

The cox regression models were adjusted for age, sex, race, body mass index, albuminuria, estimated glomerular filtration rate, hypertension, cardiovascular disease (CVD), previous stroke, smoking status, marital status, educational level, and ratio of family income to poverty.

Group 1: HbA1c ≥ 7.0%, dietary magnesium intake ≤ 250 mg/day

Group 2: HbA1c ≥ 7.0%, dietary magnesium intake > 250 mg/day

Group 3: HbA1c < 7.0%, dietary magnesium intake ≤ 250 mg/day

Group 4: HbA1c < 7.0%, dietary magnesium intake > 250 mg/day

*: p<0.05

**: p<0.01

***: p<0.001.

## Discussion

Among the population with diabetes, we demonstrated the association between higher dietary magnesium intake and lower risk of all-cause and other-cause (non-CVD, non-cancer) mortality. In addition, although individuals with baseline HbA1c ≥7.0% had higher all-cause and other-cause (non-CVD, non-cancer) mortality risk, our results showed that the association between poorly controlled diabetes and mortality risk attenuated among individuals with higher dietary magnesium intake after adjusting for potential confounders including epidemiologic factors (age, sex, and race/ethnicity), comorbidities, socioeconomic status (educational level, marital status, and family income) and behavioral factor (smoking status) (Table 3). Also, we observed that individuals with HbA1c ≥ 7% and dietary Mg > 250 mg/day (group 2) had similar mortality risk when compared with HbA1c < 7% and dietary Mg ≤ 250 mg/day (group 3). Our findings suggested that higher dietary magnesium intake may reduce the mortality risk of poorly controlled diabetes.

Magnesium is a critical cofactor to maintain adequate enzyme function in glucose metabolism, and magnesium deficiency may interfere with insulin receptor function, leading to insulin resistance [7, 17]. A previous study showed that higher dietary magnesium was inversely associated with all-cause mortality risk among a population with high cardiovascular risk, of whom less than 50% had diabetes [12]. Our study focused on individuals with diabetes and the results were consistent with previous studies in different populations [10–12]. We further showed that higher dietary magnesium intake may attenuate the mortality risk associated with poorly controlled diabetes, which has not been reported previously. Studies have shown that higher serum magnesium level may help improvement of glycemic control among diabetic patients [18, 19]. Additionally, higher total magnesium intake correlated to lower systemic inflammation and insulin resistance [20], which may lead to lower mortality risk in diabetic population as shown in our study.

Magnesium plays important roles in innate and acquired immune systems [21, 22]. In animal models, magnesium deficiency was shown to correlate to impaired function of both humoral and cell-mediated immunity, and alternative complement activation pathway was thought to be magnesium dependent [23]. On the other hand, diabetes compromises immune systems, and thus diabetic patients are susceptible to various infective diseases, which is an important cause of death in this immunocompromised population [24], especially for individuals with poor glycemic control [25]. Our study showed that higher HbA1c was associated with higher risk of death from causes other than cardiovascular disease or cancer and the risk attenuated in individuals with higher magnesium intake. We speculated that magnesium repletion may play roles in rectifying devastating immunocompromised status caused by diabetes and reduce the risk of mortality in diabetic patients.

Previous studies showed that higher serum magnesium or higher dietary magnesium correlated to lower risk of CVD events and CVD mortality in general population [10, 26, 27]. In our study, we did not observe a statistically significant association between dietary magnesium and CVD mortality risk among individuals with diabetes. However, our findings may be biased by competing risk of mortality from other causes, as evidenced by the low number of deaths from CVD. A previous study in Spain showed that magnesium intake higher than 400 mg/day correlated to lower CVD mortality but not the risk of major cardiovascular events in individuals with high risk of cardiovascular disease.^12^ Future research is needed to investigate whether higher magnesium intake could reduce the risk of death from CVD among individuals with diabetes.

Our current study demonstrated that higher dietary magnesium intake correlated to lower risk of mortality in a population with diabetes. From a public health perspective, dietary magnesium supplementation may be a cost-effective option for reducing diabetes-related morbidity and mortality. White potatoes, legumes, spinach, and whole grains are all good food sources of magnesium [28]. However, there are several study limitations that should be considered in the interpretation of our study. First, because diabetes and other comorbidities were ascertained by participants’ self-report, recall bias possibly impacts the accuracy of the measurement. Second, dietary pattern may change over time and magnesium intake may not be consistent from day to day. Similarly, single measurement of HbA1c may not exactly reflect the long-term status of diabetic control. Classifying participants by a single baseline recording could lead to misclassification bias. Third, we did not have serum magnesium data to verify the participants’ magnesium status. However, a previous study had shown that there was no correlation between serum magnesium and dietary magnesium, and that dietary magnesium but not serum magnesium was associated with insulin resistance in healthy adults [29]. Most of the total body magnesium is in the bones and soft tissues, with less than 1% of magnesium being in blood serum [30]. Therefore, serum magnesium level does not accurately reflect the participants’ magnesium status. Forth, NHANES oversampled certain subgroups of public health interest such as minorities, who may tend to have unhealthier lifestyle. In our study, we did not use survey weights, which may lead to overestimate the proportion of individuals with low dietary magnesium intake and poorly controlled diabetes. However, this would not bias the estimate of exposure and outcome association. Finally, although we have adjusted for several potential confounders, residual confounding factors may exist. For example, individuals with higher magnesium intake may have healthier lifestyle although we have controlled for several socioeconomic and behavioral factors. Given the observational nature of our study, the causal relationship between dietary magnesium intake and mortality risk cannot be confirmed.

In conclusion, our study demonstrated that higher dietary magnesium intake was associated with lower risk of all-cause and other-cause (non-CVD, non-cancer) mortality in diabetic population, while CVD and cancer mortality risk were not statistically significant probably due to competing risk of death from other causes. We also showed that mortality risk in populations with poorly controlled diabetes attenuated in those with higher dietary magnesium intake. Future research and health program intervention will be needed to elucidate the advantageous effects of dietary magnesium intake in diabetic population. In addition, we recommend including nutritional education and dietary magnesium supplementation in diabetes care practice to improve clinical outcome.

## Supporting information

S1 Table All-cause and cause-specific mortality risk associated with HbA1c ≥ 6.5% in the total population and in subgroups stratified by daily dietary magnesium (Mg) intake < and ≥ 350 mg/day. (DOCX)

S2 Table All-cause and cause-specific mortality risk among the four groups of study participants by glycohemoglobin (HbA1c) and dietary magnesium intake. (DOCX)
