# Supplementary material for: Association of dietary magnesium intake and glycohemoglobin with mortality risk in diabetic patients
Source: PLoS One. 2022 Dec 28;17(12):e0277180. doi: 10.1371/journal.pone.0277180 (PMC9797057; doi:10.1371/journal.pone.0277180)
Supplement: S1 Table — (DOCX) [file pone.0277180.s001.docx]

**Supplementary Table S1**. All-cause and cause-specific mortality risk associated with HbA1c ≥ 6.5% in the total population and in subgroups stratified by daily dietary magnesium (Mg) intake < and ≥ 350 mg/day

|  | All-cause mortality  HR (95% CI) | CVD mortality  HR (95% CI) | Cancer mortality  HR (95% CI) | Other-cause mortality  HR (95% CI) |
| --- | --- | --- | --- | --- |
| Total population |  |  |  |  |
| A1c < 6.5% (n=696) | 1 | 1 | 1 | 1 |
| A1c ≥ 6.5% (n=1,349) | 1.90 (1.30-2.78) ^***^ | 1.64 (0.75-3.57) | 1.31 (0.62-2.75) | 2.47 (1.43-4.28) ^**^ |
| Dietary Mg <350 mg/day |  |  |  |  |
| A1c < 6.5% (n=516) | 1 | 1 | 1 | 1 |
| A1c ≥ 6.5% (n=1,009) | 1.85 (1.22-2.79) ^**^ | 1.41 (0.62-3.20) | 1.22 (0.54-2.77) | 2.65 (1.41-4.99) ^**^ |
| Dietary Mg ≥350 mg/day |  |  |  |  |
| A1c < 6.5% (n=180) | 1 |  | 1 | 1 |
| A1c ≥ 6.5% (n=340) | 2.46 (0.91-6.69) | - | 2.56 (0.27-24.06) | 1.51 (0.44-5.22) |

The cox regression models were adjusted for age, sex, race, body mass index, albuminuria, estimated glomerular filtration rate, hypertension, cardiovascular disease (CVD), previous stroke, smoking status, marital status, educational level, and ratio of family income to poverty.

*: p<0.05; **: p<0.01; ***: p<0.001.
