# Supplementary material for: Association of dietary magnesium intake and glycohemoglobin with mortality risk in diabetic patients
Source: PLoS One. 2022 Dec 28;17(12):e0277180. doi: 10.1371/journal.pone.0277180 (PMC9797057; doi:10.1371/journal.pone.0277180)
Supplement: S2 Table — (DOCX) [file pone.0277180.s002.docx]

**Supplementary Table S2**. All-cause and cause-specific mortality risk among the four groups of study participants by glycohemoglobin (HbA1c) and dietary magnesium intake

|  | All-cause mortality  HR (95% CI) | CVD mortality  HR (95% CI) | Cancer mortality  HR (95% CI) | Other-cause mortality  HR (95% CI) |
| --- | --- | --- | --- | --- |
| Group 1 | 1 | 1 | 1 | 1 |
| Group 2 | 0.76 (0.47-1.21) | 0.68 (0.25-1.86) | 0.87 (0.31-2.45) | 0.74 (0.39-1.39) |
| Group 3 | 0.56 (0.37-0.84) ^**^ | 0.79 (0.35-1.77) | 0.90 (0.40-2.00) | 0.37 (0.20-0.70) ^**^ |
| Group 4 | 0.31 (0.13-0.77) ^*^ | - | 0.29 (0.04-2.22) | 0.43 (0.16-1.20) |
| *P* for trend | <0.01 | >0.05 | >0.05 | <0.01 |

The cox regression models were adjusted for age, sex, race, body mass index, albuminuria, estimated glomerular filtration rate, hypertension, cardiovascular disease (CVD), previous stroke, smoking status, marital status, educational level, and ratio of family income to poverty.

Group 1 (n=1,009): HbA1c ≥ 6.5%, dietary magnesium intake < 350 mg/day

Group 2 (n=340): HbA1c ≥ 6.5%, dietary magnesium intake ≥ 350 mg/day

Group 3 (n=516): HbA1c < 6.5%, dietary magnesium intake < 350 mg/day

Group 4 (n=180): HbA1c < 6.5%, dietary magnesium intake ≥ 350 mg/day

*: p<0.05; **: p<0.01; ***: p<0.001.
